# Development and validation of a short version of the quality of life-DSD questionnaire for parents of young children with conditions affecting sex development

**DOI:** 10.1530/EC-24-0300

**Published:** 2024-10-07

**Authors:** Salma R Ali, Melissa Gardner, Yiqiao Xin, Stuart O’Toole, Martyn Flett, Boma Lee, Mairi Steven, David E Sandberg, S Faisal Ahmed

**Affiliations:** 1 Developmental Endocrinology Research Group, Royal Hospital for Children, University of Glasgow, Glasgow, UK; 2 Office for Rare Conditions, University of Glasgow, Glasgow, UK; 3 Susan B Meister Child Health Evaluation and Research Center, Department of Pediatrics, University of Michigan Medical School, Ann Arbor, Michigan, USA; 4 Health Economics and Health Technology Assessment, Institute of Health and Wellbeing, University of Glasgow, Glasgow, UK; 5 Department of Paediatric Surgery, Royal Hospital for Children, Glasgow, UK

**Keywords:** health-related quality of life, parent/patient-reported outcome, psychosocial screening

## Abstract

**Background:**

There is a paucity of information on health-related quality of life (HRQoL) outcomes in parents and children with conditions affecting sex development. The objective of this study was to develop short forms of HRQoL questionnaires which consist of a 63-item and 25-item parent self-report (PSR) and parent proxy-report (PPR), respectively, optimizing use in routine clinical settings.

**Methods:**

Short questionnaires were developed following exploratory factor analysis using raw data from 132 parents. Long and short PSRs were completed by 24 parents of children with conditions affecting sex development, with a median age of 3.6 years (range 0.1, 6.6); 21 (88%) were boys, and 11 (46%) had proximal hypospadias. A subset of 19 parents completed both long and short PPRs.

**Results:**

Item selection, based on factor loadings of >0.8 and expert consultation, produced short PSRs and PPRs containing 16 and 7 items, respectively. There was no statistically significant difference in 11 out of 12 (92%) scales on the PSR and 4 out of 5 (80%) scales on the PPR when comparing short and long questionnaire scores. The short and long questionnaires took <1 min and 5 min to complete, respectively. Eighteen parents (75%) reported that the time taken to complete the short questionnaires was acceptable; 10 (42%) preferred short questionnaires. Ten (42%) versus 6 (25%) stated a preference for completing the short versus long questionnaires.

**Conclusion:**

The short versions were largely representative of the long questionnaires and are acceptable for evaluating psychosocial distress in young children and their caregivers. Further psychometric validation of the short forms is warranted.

## Introduction

Conditions affecting sex development include a wide range of conditions that most often present in infancy or in the adolescent period, are characterized by atypical chromosomal, gonadal, or phenotypic sex (1) and may be associated with adverse psychosocial and psychosexual outcomes (2, 3, 4, 5, 6). Studies have shown that a subset of parents of children with such conditions can experience high levels of depression, anxiety, and stress related to concerns regarding their child’s future (7, 8, 9, 10). However, there is relatively little information regarding psychosocial functioning and health-related quality of life (HRQoL) outcomes in parents and young children (11, 12, 13), and there is a lack of validated instruments available for evaluating these outcomes longitudinally in a routine clinic setting.

Recent studies have shown that caregivers’ perceptions of uncertainty regarding their child’s condition are highest soon after the initial presentation, which is frequently prenatally or soon after birth (11). This illness uncertainty may continue to predict both anxiety and depressive symptoms over time (14, 15, 16, 17, 18). Thus, longitudinal monitoring of health and quality of life status may help in the early identification of parental and child distress, providing opportunities for timely intervention and psychosocial support for both parents and children. In addition, the development of condition-specific HRQoL assessment tools may guide clinical decision-making, inform changes in practice, and enable the evaluation of outcomes.

Until recently, there were no standardized measures to capture parental stressors in children with conditions affecting sex development. In 2016, HRQoL questionnaires were developed for parents of young children with disorders or differences of sex development (QoL-DSD) (19), in accordance with the Food and Drug Administration’s guidelines on the development of patient-reported outcome (PRO) measures (20). The questionnaire items were derived from focus groups and interviews involving clinicians with DSD expertise and parents of children with a disorders of sexual development (DSD). The parent self-report (PSR) questionnaire (for parents of children aged from birth to under 7 years) comprised of 63 items (within 13 scales). The PSR assessed caregiver feelings and experiences in relation to decision-making, healthcare communication, role functioning and family activities, gender concerns, socio-emotional functioning, future concerns, administration of medication, surgical procedures, doctor’s visits, and talking to others regarding their child’s DSD condition. The parent proxy report (PPR) (for parents of children aged 2 to under 7 years) comprised 25 items (within five scales) and was a caregiver’s report of their child’s feelings and experiences regarding physical functioning, gender concerns, socio-emotional functioning, and doctor’s visits in relation to their DSD diagnosis (19).

The length of HRQoL instruments is often perceived as a barrier against their implementation in routine clinical practice, and a shorter questionnaire may broaden its application by reducing the burden of data collection and the risk of item non-response. As long as the content and domains of the original longer instrument can be reflected without unduly affecting the instrument’s psychometric properties, it is often recommended that an abbreviated version of the instrument should also be explored (21). The aims of this study were to develop short versions of the PSR and PPR QoL-DSD questionnaires, perform initial validation of the shortened questionnaires, and determine their acceptability of the questionnaires within a cohort of parents of children with conditions affecting sex development.

## Methods

### Development of short questionnaires

To select items for inclusion in the short versions of the parent self-report and parent proxy-report questionnaires (Short PSR and Short PPR), we utilized raw data from 132 participants who had previously completed the long versions of the QoL-DSD questionnaires (Long PSR and Long PPR) as part of a validation study (19). The original long questionnaires consist of a 63-item PSR and a 25-item PPR questionnaire to be completed by parents of children aged from birth to 7 years and children aged 2 to 7 years, respectively. Exploratory factor analysis (EFA) with maximum likelihood and varimax rotation was performed. To maintain the original scale structure of the PSR and PPR long questionnaires, each scale was analyzed separately, and items with factor loadings (FLs) of >0.8 were selected for consideration of inclusion in the short questionnaires. Clinical reasoning decisions via consultation with expert healthcare staff, including pediatric endocrinologists from the Department of Child Health, Glasgow, and pediatric clinical psychologists from the Child Health Evaluation and Research Center, Michigan, were incorporated to decide upon the items for inclusion in the short questionnaires.

### Participant selection

Parents or caregivers of children from birth to 7 years with DSD, attending an endocrine or urology clinic at the Royal Hospital for Children, Glasgow, between April 2021 and March 2022, were recruited. Eligibility was restricted to those with sufficient fluency and literacy in English, such that they did not require a translator. One caregiver completed the questionnaires per family. The study was approved by the West of Scotland Research Ethics Committee, and informed consent was obtained from all participants.

### Administration of questionnaires

Participants were asked to complete short and long versions of questionnaires on a single occasion. PSR questionnaires were completed by parents of children aged from birth to 7 years old, among whom those of children aged 2 years or above also completed the PPR questionnaires. A computer-generated randomization sequence using Microsoft Excel was applied to determine questionnaire allocations to participants and whether the short or long versions were completed first. Following non-completion of questionnaires, a reminder email was sent once to caregivers within a 2-week period. All questionnaires and the feedback form were developed and hosted online via Webropol (https://webropol.com/), a secure online platform endorsed and supported by NHS Greater Glasgow & Clyde and NHS Scotland. Questionnaires and feedback forms were completed by caregivers in their home setting. All information within Webropol is kept in compliance with the UK Data Protection Act (2018) and General Data Protection Regulation (GDPR 2016/679).

### Parental feedback

Participant feedback was evaluated immediately following the completion of the online questionnaires. Participants rated their experience on a five-point Likert scale (ranging from ‘strongly agree’ to ‘strongly disagree’) regarding the ease of completing both short and long questionnaires, their comprehension, the time required for completion, and their preference (short/long/both/neither) regarding the questionnaires.

### Questionnaire scoring and statistical analysis

Scoring options were kept consistent within long and short versions of the PSR and PPR questionnaires. Response options were categorized on a five-point Likert scale assessing the level of agreement (always true/usually true/sometimes/seldom true/never true) or distress (a great deal/moderate/some/a little/none) in relation to questionnaire items. Not applicable (N/A) response options were available for items relating to surgery, medication, or if the child was deemed too young to seek a response (e.g. talking with your child before a doctor’s visit). Items within each subscale were assigned values ranging from 0 to 4. A scale average was obtained, divided by 4 (the highest possible score), and multiplied by 100 to obtain an overall score for each scale. For each scale, a value ranging from 0 to 100 was obtained, with 100 representing the best (most optimal) score and indicating better quality of life. If a response of not applicable was provided, it was not included in the score. The median difference in overall scale scores between the short and long questionnaires among participants was determined and analyzed using the Wilcoxon signed-rank test. A *P* value of <0.05 was considered statistically significant. Concurrent validity was determined by the amount of agreement between the same items within the short questionnaires and the corresponding scales on the long questionnaires, using Pearson’s correlation coefficient.

## Results

### Development of short versions of PSR questionnaire

Following EFA, 18 items on the Long PSR had FL >0.8 (Table 1). Of these 18 items, 13 items were selected for inclusion in the Short PSR, along with an additional three items that had FL <0.8, however, were selected based on clinical reasoning. The items selected based on clinical reasoning were: i) ‘I worry about talking to others about my child’s condition because of how they might react’(FL, 0.7); ii) ‘I am concerned about how my child’s genitals look’ (FL, 0.68); and iii) ‘Thinking about your child’s last doctor’s visit for his/her condition, how much stress did you experience not knowing what to expect at the visit’ (FL, 0.6). The five items with FLs >0.8 that were removed included an item in the decision-making scale about having enough information to understand the child’s needs; an item in the gender concerns scale about caregiver confidence that their child feels comfortable with their gender; and future concerns regarding the child’s genital function. These items were subsumed by other items within these scales. Addtionally, two items in the healthcare communications and information scale, which corresponded with healthcare service evaluation, were also removed.

**Table 1:** Parent self-report items on the Long QoL-DSD questionnaire and factor loadings.

Scales and corresponding items	Factor loading^a^
*Decision-making*	
Thinking about the past 2 weeks, how true are these statements for you:	
A1. I have enough information about my child’s condition to understand what his/her needs are	0.89
A2. I have enough information about my child’s condition to make decisions about his/her care	0.94
A3. I feel confident I made the best decisions about surgery for my child (including not having surgery)	0.36
A4. I agree with my child’s doctor’s current recommendations about care	0.53
*Role functioning & family activities*	
In the past 2 weeks, how much stress did you experience:	0.62
**B6. Fitting your child’s care for his/her condition into your usual routines or daily activities**	**0.87**
B7. Helping your child with toilet training or using the bathroom due to his/her condition	0.64
B8. Being protective of your child because of his/her condition	0.61
*Gender concerns*	
Thinking about the past 2 weeks, how true are these statements for you:	
**C9. I am confident my child’s gender was identified correctly**	**0.84**
C10. I feel comfortable with my child’s gender behaviors (e.g. play interests, toy, and playmate choices)	0.59
C11. I am confident I am raising my child in the right gender	0.66
**C12. I am confident my child feels comfortable with his/her gender**	**0.87**
*Social functioning*	
Thinking about the past 2 weeks, how true are these statements for you:	
D13. I feel disconnected from my family because of my child’s condition	0.74
**D14. My child’s condition affects how often I go out socially**	**0.87**
D15. I socialize with family and friends as much as I would like to	0.43
*Emotional functioning*	
Thinking about the past 2 weeks, how true are these statements for you:	
**E16. I feel happy**	**0.99**
E17. I have difficulty sleeping at night	0.06
E18. I feel irritable	0.45
E19. I enjoy social activities	0.49
E20. I feel overwhelmed	0.41
E21. I feel upset	0.50
*Future concerns*	
How true are these statements for you:	
F22. I am concerned about how my child’s genitals will look	0.76
**F23. I am concerned about how my child’s genitals will function**	**0.82**
F24. I worry about my child dying due to the condition and/or its treatment	0.42
F25. I worry that my child will have fertility issues (e.g., will not be able to have a biological child)	0.73
**F26. I feel concerned my child will have social problems, like being teased about his/her condition**	**0.80**
**F27. I worry about my child’s future relationships (e.g., dating, marriage)**	**0.82**
F28. I worry that my child will not be comfortable with his/her gender as an adult	0.37
*Healthcare communication & information*	
Please answer the following questions while thinking about the present, even if your responses might have been very different in the past.	
G29. My child’s doctors are knowledgeable about my child’s condition	0.68
G30. I feel overwhelmed by the amount of information about my child’s condition	0.33
**G31. My child’s doctors are sensitive (i.e. supportive, considerate) when communicating with me about my child’s condition**	**0.85**
G32. I hear conflicting medical information about my child’s condition	0.62
**G33. My child’s doctors explain everything clearly**	**0.89**
G34. I feel stressed when dealing with health insurance and/or medical costs	0.21
*Talking to others*	
How true are these statements for you currently:	
**H35. I feel comfortable talking with my child about his/her condition**	**0.92**
H36. I feel comfortable talking to close family members about my child’s condition	0.57
H37. I am comfortable explaining my child’s needs (e.g., diaper changing, using the bathroom) to people other than family	0.63
H38. I am not sure how much to tell others about my child’s condition	0.70
H39. I worry about talking to others about my child’s condition because of how they might react	0.76
*Medications*	
Thinking about the past 2 weeks, how much stress did you experience:	
If your child does not take any medications for his/her condition, please go to the next section (J).	
I40. Remembering to give your child his/her medications related to the condition (e.g. hormones such as cortisone, prednisone, or testosterone)	0.67
**I41. Making sure your child receives his/her medications for the condition when he/she is away from you (e.g., at school or daycare)**	**0.82**
I42. Giving your child his/her medication	0.76
I43. Managing the side effects of your child’s medication	0.59
*Surgery*	
Thinking about your child’s last surgery for his/her urogenital condition, how much stress did you experience:	
If your child has not had any surgeries, please go to the next section (K).	
J44. Talking with your child before the surgery	0.39
**J45. During the surgery**	**0.98**
J46. After the surgery (e.g. dealing with your child’s need for extra care, wondering about the outcome)	0.61
*Doctor’s visits*	
Thinking about your child’s last doctor’s visit for his/her condition, how much stress did you experience:	
K47. Talking with your child before the visit	0.74
K48. Not knowing what to expect at the visit	0.60
**K49. Managing your child’s behavior during the visit**	**0.85**
*Earliest experiences*	
Thinking back to the time when your child’s condition was first noticed, how much stress did you experience:	
L50. Not knowing whether your child was a boy or a girl	0.66
L51. Waiting for your child’s diagnosis	0.72
**L52. Receiving your child’s diagnosis**	**0.88**
L53. Thinking about how this condition might affect your child	0.79
L54. Thinking about how this condition might affect you and your family	0.70
*Clinical items*	
Thinking about the past two weeks, how true are these statements for you:	
M1. I am concerned about how my child’s genitals look	0.68
M2. Because of the condition, I have disagreements with family members about whether I should raise my child as a typical boy or girl	0.03
**M3. I worry I could have another child with the same condition**	**1.00**
M4. I feel comfortable talking to my partner/spouse about my child’s condition	0.26

^a^Questionnaire items with factor loadings >0.8 are highlighted in bold.

### Development of short versions of PPR questionnaire

Following EFA, six items on the Long PPR had FL >0.8 (Table 2). Of these six items, four items were selected for inclusion in the Short PPR, and an additional three items were selected based on clinical reasoning. The items selected based on clinical reasoning were: i) ‘My child experiences physical pain when urinating’ (FL 0.69); ii) ‘My child has concerns about going to a public restroom because of his/her condition’ (FL 0.62); and iii) ‘Thinking about the past 2 weeks, how much stress does your child experience taking medication for his/her condition?’. Two items with FLs >0.8 in the gender concerns (‘My child has commented on being unhappy with how his/her genitals look and function’) and medical care (‘Thinking about your child’s last doctor’s visit or medical procedure, how much stress did your child experience having doctors examine his/her genitals’) scales were omitted, as these were subsumed by other selected items within these scales.

**Table 2:** Parent proxy-report items on the Long QoL-DSD questionnaire and factor loadings.

Scales and corresponding items	Factor loading^a^
*Physical functioning*	
Thinking about the past 2 weeks, how true are these statements for your child:	
A1. Due to his/her condition, my child experiences physical pain when urinating	0.69
A2. My child experiences physical pain due to his/her condition other than during urination	0.25
A3. My child urinates differently than other children of his/her same gender (e.g. where urine comes from)	0.13
A4. Due to his/her condition, my child has difficulty with toilet training (if toilet trained: he/she has difficulty using the bathroom)	0.25
**A5. My child’s condition affects his/her activities (e.g. play dates, swimming, sports)**	**0.96**
*Gender concerns*	
B6. My child’s play interests match his/her gender	0.45
B7. My child’s playmate choices match his/her gender	0.49
**B8. My child has commented on being unhappy with how his/her genitals look and function**	**0.92**
**B9. My child feels different from other children of the same gender due to his/her condition**	**0.94**
*Socio-emotional functionin*g	
Thinking about the past 2 weeks, how true are these statements for your child:	
C10. My child has concerns about going to a public restroom because of his/her condition	0.62
C11. My child is able to make friends	0.43
C12. My child adjusts well to new social situations	0.72
C13. My child seems happy	0.59
C14. My child gets into fights with other children (e.g. with siblings or peers)	0.37
C15. My child has as much fun as other children	0.80
C16. My child seems withdrawn	0.69
**C17. My child has more difficulty being away from parents than other children his/her age**	**0.84**
*Medical care*	
Thinking about your child’s last doctor’s visit or medical procedure, how much stress did your child experience:	
D18. Before the doctor’s visit or procedure (e.g. on the way to	0.65
the appointment, in the waiting room)	
**D19. Having doctor’s visits (e.g. physical exams)**	**0.95**
**D20. Having doctors examine his/her genitals**	**0.85**
D21. Having medical procedures (e.g. blood draws)	0.56
D22. After the visit or procedure	0.56
*Clinical item*	
Thinking about the past two weeks, how much stress does your child experience:	
E1. Taking medication for his/her condition	**-**

^a^Questionnaire items with factor loadings >0.8 are highlighted in bold.

### Participant characteristics

**Figure 1:**
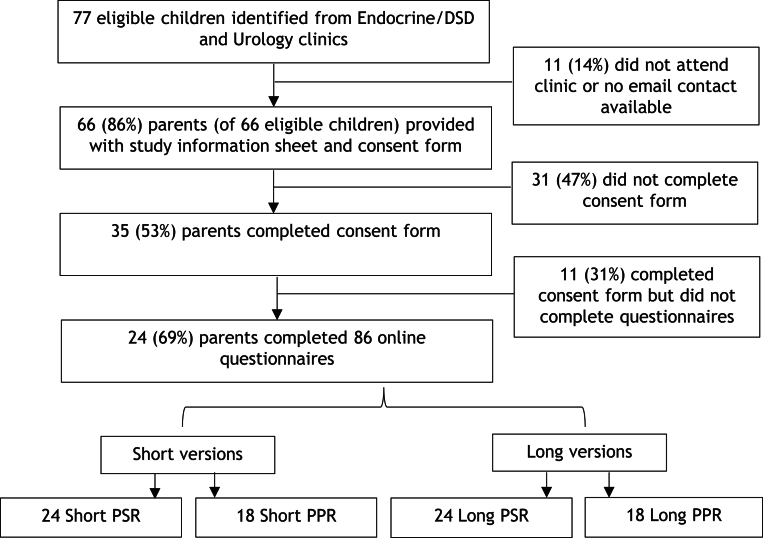
Flow diagram of response rates.

Of the 66 caregivers approached, 24 (36%) completed a total of 86 questionnaires (Fig. 1, Table 3), including 48 Short and Long PSRs and 36 Short and Long PPRs. The median age of the children was 3.6 years (range 0.1, 6.6), and of the 24 children, 21 (88%) were boys and 3 (12%) were girls. Of the 21 boys, 11 (46%) had proximal hypospadias, five (21%) had distal hypospadias, and five (24%) had other DSD diagnoses, including partial gonadal dysgenesis (*n* = 1), bilateral undescended testes (*n* = 1), hypogonadotropic hypogonadism (*n* = 1), Klinefelter syndrome (*n* = 1), and congenital adrenal hyperplasia (CAH) (*n* = 1). All three girls had a diagnosis of CAH.

**Table 3:** Participant characteristics.

Caregivers	*n*, %
Parents	24
Mother	19 (79%)
Father	4 (17%)
Other (foster carer)	1 (4%)
Age, years	
16–24	1 (4%)
25–34	9 (38%)
35–44	12 (50%)
45–54	1 (4%)
Prefer not to answer	1 (4%)
Parent race	
Caucasian	20 (83%)
Asian	4 (17%)
Marital/partnership status	
Never married and never registered in a civil partnership	3 (13%)
Married	17 (71%)
In a registered civil partnership	2 (8%)
Prefer not to answer	2 (8%)
Highest level of education	
Early childhood education	1 (4%)
Secondary education	5 (21%)
Tertiary education	6 (25%)
Bachelor’s degree or equivalent	6 (25%)
Master’s degree or equivalent	3 (13%)
Prefer not to answer	2 (8%)
Level of education not classified	1 (4%)
Children	24
Gender	
Girl	3 (12%)
Boy	21 (88%)
Median age, years	
0–1.9 years	5 (21%)
2–4.9 years	11 (46%)
5–7 years	8 (33%)
DSD diagnosis	
Proximal hypospadias	11 (46%)
Distal hypospadias	5 (21%)
Congenital adrenal hyperplasia	4 (17%)
Partial gonadal dysgenesis	1 (4%)
Bilateral undescended testes	1 (4%)
Hypogonadotrophic hypogonadism	1 (4%)
Klinefelter syndrome	1 (4%)

### Validation of questionnaires (short versus long forms)

**Figure 2:**
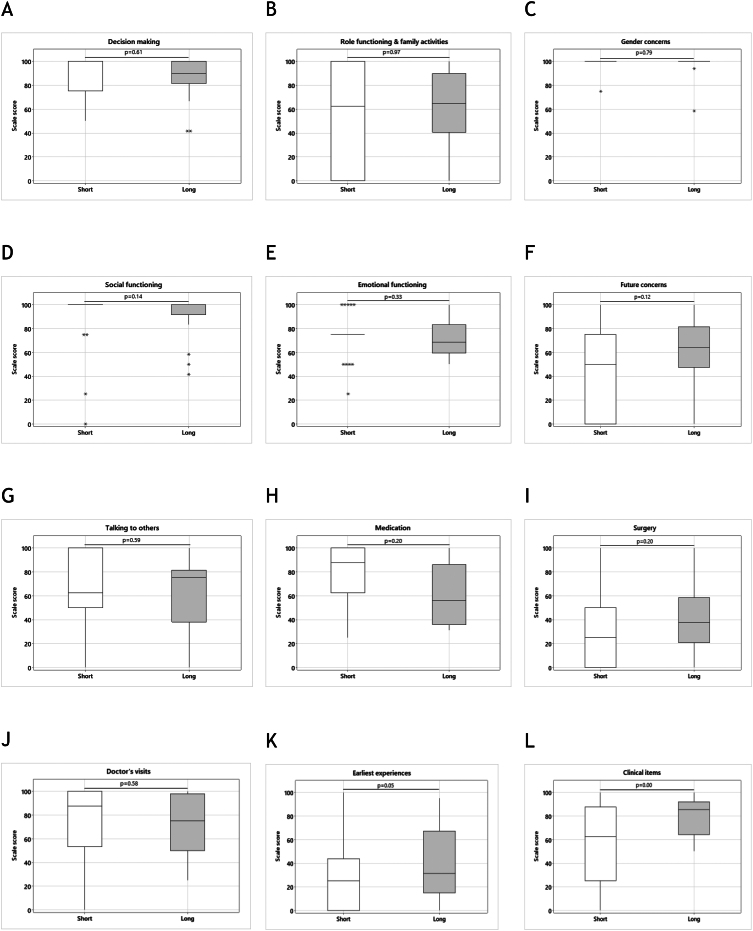
Comparison of overall scale scores for the quality of life – differences of sex development short parent self-report questionnaires (Short PSR) and the quality of life – differences of sex development long parent self-report questionnaires (Long PSR).

**Figure 3:**
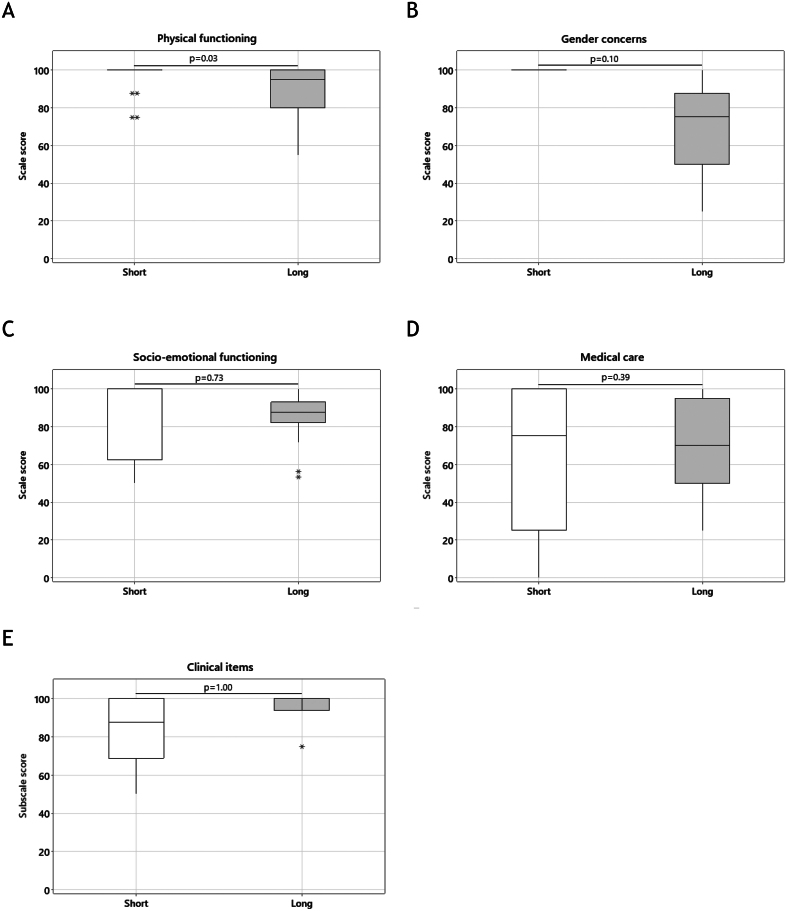
Comparison of overall scale scores for quality of life – differences of sex development short parent proxy-report questionnaires (Short PPR) and quality of life – differences of sex development long parent proxy-report questionnaires (Long PPR).

A total of 16 items within 13 scales were included in the Short PSR, and a total of seven items within five scales were included in the Short PPR. The overall scale scores for the 12 scales on the Short PSR were compared with the concurrent scales on the Long PSR (Figs 2 and 3). There were no statistically significant differences in overall scale scores for 11 out of 12 (92%) scales (all *P* > 0.05), with a significant difference noted only in the clinical items scale (*P* < 0.001). In addition, the overall scale scores for the five scales on the Short PPR were compared with the concurrent scales on the Long PPR. When comparing the Short PPR with the long version, there were no differences in overall scale scores for 4 out of 5 (80%) scales (all *P* > 0.05), with a significant difference noted only in the physical functioning scale (*P* = 0.03) (Figs 2 and 3). Correlations between scale scores on the Short and the Long questionnaires ranged from 0.50 to 0.89 in 9 out of 12 scales on the PSR questionnaire and were 0.60 in 2 out of 5 scales on the PPR questionnaire (Supplementary Table 1, see section on supplementary materials given at the end of this article).

### Parental views

Of the 24 parents, 17 (70%) and 18 (75%), respectively, agreed that the short and long versions of the questionnaires, were easy to understand. The time taken to complete the short questionnaire was less than 1 min, while the long questionnaire took around 5 min. Eighteen (75%) and 16 (67%) parents, respectively, agreed that the time taken for completion of the short and long forms was acceptable; of these, 12 (50%) versus 9 (38%), respectively, strongly agreed. Ten (42%) parents preferred the short version of the questionnaires, compared with five (21%) who preferred the long version. Ten (42%) parents stated a preference for completing the short forms in the future should these be implemented as a routine screening tool, compared with six (25%) who stated a preference for the long versions.

## Discussion

This is the first study to report the development and the first phase of validation of a short PRO questionnaire that could be used in the routine clinical settings to screen for HRQoL problems among parents and caregivers of young children with conditions affecting sex development, including DSD.

Item reduction resulted in significantly shortened versions of the longer questionnaires, with a 16-item short version of the PSR and a seven-item short version of the PPR. Scores on the short forms were compared with those on the long forms, which had been validated in a previous study (19). Our results showed that the short questionnaires were largely representative of the long questionnaires. There were no significant differences in the overall scale scores when comparing the short with long versions in all but one scale of the PSR questionnaire. In addition, there were no significant differences in the overall scale scores when comparing the short with long forms in all but one scale of the PPR questionnaire.

The differences in overall scale scores in the clinical items and physical functioning scales of the PSR and PPR questionnaires, respectively, may be attributed to the reduced number of items included within the final scales of the short form. Varied themes within select items resulting in different responses to items within each scale and the applicability of questions in relation to individual DSD conditions may also account for these differences. Furthermore, the age applicability of specific questions is another possible factor, and almost half of the children for whom the proxy reports were completed were aged 2–4.9 years. Within the physical functioning scale of the short PPR, 2 out of 5 items were selected for inclusion. These included one item on physical pain experienced when urinating and a second item on the effect of the child’s condition on his/her activities (e.g. play dates, swimming, sports). The long version of the questionnaire had five items in the latter scale, with 4 out of 5 items addressing urinary problems (pain, urinating differently from other children, difficulty with toilet training). Thus, the differences in this scale may be attributed to both the applicability of the items in relation to the particular condition and age of the child. All items from the two scales where a statistically significant difference between the short and long forms was identified will be included in the final version of the short questionnaires, which will undergo further validation in a larger cohort.

Respondent time burden was significantly reduced with short questionnaires, and three-quarters of caregivers agreed that the short questionnaires had an acceptable time for completion. Overall, almost half of caregivers stated a preference to complete the short questionnaires compared with less than a quarter who stated a preference for the longer versions. In addition, almost half of caregivers stated a preference to complete the shorter questionnaires should these be implemented as a routine screening tool in the future, suggesting that short versions may be more acceptable for use in a routine outpatient setting.

The demographics of the original sample of 132 parents (19) were similar to our validation sample in terms of the proportion of mothers completing questionnaires, the parental degree-level education, the proportion of male patients, and the diagnosis. A direct comparison between the short and long questionnaires was facilitated by ensuring that the questions were consistent with the previously validated long questionnaires and that the response options were categorized on a five-point Likert scale. Retaining the same scale structure as the original questionnaires also ensured the preservation of the themes identified as important through previous research and focus groups (19). Another potential advantage of multi-item scales within questionnaires is more reliable findings and a greater distribution of responses for capturing varying degrees of satisfaction. Short questionnaires offer ease of administration and data entry by participants. It may also be the case that if concerns are identified via the short questionnaires in a routine clinic setting, the provision of longer, more detailed questionnaires could be administered subsequently, allowing more targeted interventions including psychology input to improve outcomes. The current study suggests that parents would be willing to complete the long questionnaire if necessary. The inclusion of participant demographic information in the questionnaires, including the relationship of the caregiver to the child, caregiver age, and educational status, was also important. Previous studies have reported that mothers may experience greater levels of distress in relation to their child’s DSD condition (12, 22, 23) and that the level of decisional regret (distress or remorse after a healthcare decision) was related to factors including having a higher level of education, increased levels of illness uncertainty preoperatively, and persistent illness uncertainty at 12 months after genitoplasty for atypical genitalia (14, 24). In our study, there was a preponderance of Caucasian participants (83% of the cohort). Previous studies have reported that factors such as ethnicity may influence the willingness to complete surveys, with higher non-response rates among ethnic minorities, however it remains unclear whether they influence the survey results. Further study is needed to investigate this aspect within the context of the wide range of conditions that can affect sex development (25).

Some limitations were also noted. Within the short and long questionnaires, some items within the scales had non-applicable response options and these were not included in the overall scale scores. Some children were not taking medication, had not undergone previous surgery for their condition, or were too young for responses to be provided; thus, the overall number of responses for individual scales was variable. There was also the potential for individuals completing these questionnaires to omit items or become confused by being asked similar questions phrased in a different manner, resulting in a reduction in reliability. Nevertheless, exploring potential multiple dimensions of a single concept is important.

Further assessment of the psychometric properties of the short questionnaires in a larger cohort, including test–retest reliability and group construct validity, will result in reliable and valid measures, facilitating their use in routine clinical settings. Performing future PRO studies in various languages across international settings and among older age groups could provide valuable insights into potential differences in psychosocial outcomes across samples from diverse social and cultural backgrounds. By using the data that will be generated, it will be possible to investigate the extent of psychosocial distress experienced by young children with conditions affecting sex development and their families, implement early psychosocial interventions, and establish clinical benchmarks for these outcomes.

In conclusion, this study has created short forms of a recently validated set of questionnaires for the assessment of HRQoL for parents of young children with a condition that may affect sex development. These short questionnaires may have utility in routine clinical practice but require further validation.

## Supplementary Materials

Supplementary Table 1. Items in the short questionnaires and corresponding scales on the QoL-DSD.

## Declaration of interests

SFA is the Editor-in-Chief of Endocrine Connections. SFA was not involved in the review or editorial process for this paper, on which he is listed as an author.

## Funding

SRA was supported by an unrestricted education grant from Diurnal, a small project grant from the Glasgow Children’s Hospital Charity, and the Gardiner Lectureship at the University of Glasgow; DES and MG were supported by grants R01 HD053637 and R01 HD093450 from the Eunice Kennedy ShriverNational Institute of Child Health and Human Developmenthttp://dx.doi.org/10.13039/100000071.

## Ethics approval and consent to participate

The study protocol followed the ethical principles of the Helsinki Declaration and was approved by the West of Scotland Research Ethics Committee. Written informed consent was obtained from all participants.

## Data availability

The data that support the findings of this study are available from the corresponding author upon reasonable request.

## Author contribution statement

SRA: Conceptualization (lead), Data curation (lead), Formal analysis (lead), Funding acquisition (support), Investigation (lead), Methodology (lead), Project administration (lead), Writing – original draft (lead), Writing – review & editing (lead]); MG: Conceptualization (equal), Data curation (supporting), Methodology (supporting), Writing – original draft (supporting), Writing – review & editing (supporting)); YY: Methodology (supporting), Writing – review & editing (supporting)); SO’T: Writing – review & editing (supporting)); MF: Writing – review & editing (supporting)); BL: Writing – review & editing (supporting)); MS: Writing – review & editing (supporting)); DES: Conceptualization (equal), Data curation (supporting), Methodology (supporting), Supervision (supporting), Writing – original draft (supporting), Writing – review & editing (supporting)), and SFA: Conceptualization (equal), Data curation (supporting), Funding acquisition (lead), Methodology (supporting), Supervision (lead), Project Administration (supporting), Resources (lead), Writing – original draft (supporting), Writing – review & editing (supporting)).

## Acknowledgements

The authors would like to thank all patients and caregivers who agreed to participate in this study.
