# Supplementary material for: Development and validation of a short version of the quality of life-DSD questionnaire for parents of young children with conditions affecting sex development
Source: Endocr Connect. 2024 Oct 7;13(11):e240300. doi: 10.1530/EC-24-0300 (PMC11466247; doi:10.1530/EC-24-0300)
Supplement: Supplementary Table 1. Items in the short questionnaires and corresponding scales on the QoL-DSD. [file supplementary_table_1.pdf]

Supplementary Table 1. Items in the short questionnaires and corresponding scales on the QoL-DSD.

**Parent self-report**			
16 Items on short version (Short PSR)	Corresponding scales on long version (Long PSR)	*n*	*r*
• I have enough information about my child's condition to make decisions about his/her care	Decision Making	21	0.50
• Fitting your child's care for his/her condition into your usual routines or daily activities	Role Functioning & Family Activities	24	0.23
• I am confident my child's gender was identified correctly	Gender Concerns	24	0.05
• My child's condition affects how often I go out socially	Social Functioning	24	0.37
• I feel happy	Emotional Functioning	24	0.71
• I feel concerned my child will have social problems, like being teased about his/her condition<br>• I worry about my child's future relationships (e.g., dating, marriage)	Future Concerns	24	0.70
• I feel comfortable talking with my child about his/her condition<br>• I worry about talking to others about my child's condition because of how they might react	Talking to Others	24	0.72
• Making sure your child receives his/her medications for the condition when he/she is away from you (e.g., at school or daycare)	Medications	5	0.74
• During the surgery	Surgery	13	0.60
• Not knowing what to expect at the visit<br>• Managing your child's behaviour during the visit	Doctor's Visits	24	0.89
• Receiving your child's diagnosis	Earliest Experiences	24	0.76
• I am concerned about how my child's genitals look<br>• I worry I could have another child with the same condition	Clinical Items	24	0.80
**Parent proxy-report**			
7 Items on short version (Short PPR)	Corresponding scales on long version (Long PPR)	*n*	*r*
• Due to his/her condition, my child experiences physical pain when urinating<br>• My child's condition affects his/her activities (e.g., play dates, swimming, sports)	Physical Functioning	19	0.34
• My child feels different from other children of the same gender due to his/her condition	Gender Concerns	5	n/a
• My child has concerns about going to a public restroom because of his/her condition<br>• My child has more difficulty being away from parents than other children his/her age	Socio-Emotional Functioning	9	0.56
• Having doctor's visits (e.g., physical exams)	Medical Care	19	0.57
• Taking medication for his/her condition	Clinical Item	5	0.25

Note. n's vary as some children were not taking medication, had not had previous surgery or were too young for responses to be provided. *r*; Pearson's correlation coefficient.
